# The prevalence of non-sentinel lymph node metastasis among breast cancer patients with sentinel lymph node involvement and its impact on clinical decision-making: a single-centred retrospective study

**DOI:** 10.3389/or.2024.1495133

**Published:** 2024-10-31

**Authors:** Jingxian Ding, Xiaoliu Jiang, Zhaohui Huang, Qiao Ji, Jie Long, Yali Cao, Yonghong Guo

**Affiliations:** ^1^ Department of Radiation Oncology, The Breast Cancer Institute, Nanchang People’s Hospital, Nanchang, Jiangxi, China; ^2^ Department of Breast Surgery, The Breast Cancer Institute, Nanchang People’s Hospital, Nanchang, Jiangxi, China; ^3^ Department of Radiation Oncology, Affiliated Rehabilitation Hospital of Nanchang University, Nanchang, Jiangxi, China

**Keywords:** breast cancer, lymph node metastasis, sentinel lymph node biopsy, axillary lymph node dissection, radiotherapy

## Abstract

**Background:**

Sentinel lymph node biopsy (SLNB) has become standard procedure for early breast cancer patients with clinically node negative disease. The patients with SLN metastasis normally underwent axillary lymph node dissection (ALND). However, the metastatic status of non-sentinel Lymph nodes (non-SLNs) varied significantly in different reports. Here, we evaluated the prevalence of non-SLNs metastasis among breast cancer patients with sentinel lymph node metastasis and its impact on clinical decision-making.

**Materials and Methods:**

We identified 892 female patients with operable cT1-3N0 invasive breast cancer who underwent ALND in our center due to SLN metastasis from 2017 to 2023, retrospectively. The prevalence of non-SLN metastasis among different clinicopathological traits and its correlation with the number of positive SLNs were analyzed. The optimal clinical decision-making was generalized.

**Results:**

The median number of SLN+, SLN, non-SLN+ and non-SLN was 2, 4, 1 and 14 among the enrolled 892 female patients, respectively. 504 (56.50%) patients with SLN + had at least one metastatic lymph node in the harvested non-SLNs. Among the enrolled 892 female patients, 435 (48.77%) patients with 1 positive SLN, of which 180 (41.38%) had at least one additional metastatic non-SLNs. 242 (27.13%) patients with 2 positive SLNs, of which 146 (60.33%) had at least one metastatic non-SLNs. For the rest 215 (24.10%) patients with at least 3 metastatic SLNs, 178 (82.79%) had at least one metastatic non-SLNs. In the univariate analysis, the non-SLNs metastatic status was correlated with the number of SLNs+, tumor size, tumor grade, lymphovascular invasion (LVI) and molecular subtypes, but not histopathologic type. In the multivariate analysis, the risk of additional non-SLNs metastasis correlated with the number of SLNs+, SLNs, non-SLNs and LVI.

**Conclusion:**

Omiting ALND in patients with higher non-SLNs + rate outside the American College of Surgeons Oncology Group (ACSOG) Z0011 and the European Organization for Research and Treatment of Cancer (EORTC) 10,981–22023 AMAROS criteria should be considered with caution in clinical decision-making. To evaluate whether axillary radiotherapy and ALND provides equivalent regional control in breast cancer patients with obvious residual metastatic lymph nodes undesected in the axilla, a well-matched prospective randomized controlled trial is an urgent need.

## Introduction

The breast cancer is one of the most frequently diagnosed cancers and the fifth most common cause of cancer related death among women worldwide (1, 2). Its prognosis has improved due to the tremendous efforts made in the early detection and the development of treatment modalities including surgical techniques and adjuvant treatments post-surgery, such as endocrine therapy, chemotherapy, human epidermal growth factor receptor type 2 (HER2) targeted therapy and radiotherapy (3, 4, 5, 6). Patient quality of life (QoL) is increasingly being highly valued. Axillary lymph node metastasis is an important factor in determining the stage of breast cancer and deciding postoperative treatment, which is an independent prognostic factor associated with local or distant metastatic recurrence (7). Therefore, to precisely evaluate axillary lymph node status is essential for the standardized diagnosis and treatment of breast cancer, and axillary lymph node dissection (ALND) has been applied in surgical treatment of breast cancer for several decades. However, postoperative lymphedema of the upper limb often puzzles patients with breast cancer after ALND (8). Lymphedema is a chronic disease characterized by an accumulation of lymphatic fluid, resulting in skin and tissue changes (9). Breast cancer-related lymphedema develops as a result of damage or dysfunction of the normally functioning lymphatic system mainly due to ALND, which significantly impacts patient QoL (8, 10, 11). Therefore, it is very important to preserve the armpit for improving the QoL of breast cancer patients, especially for early breast cancer patients with clinically negative axillary lymph nodes. Over 2 decades ago, the sentinel lymph node biopsy (SLNB) was introduced into axillary staging in operable early breast cancer (12). SLNB was originally used for cT1-2 disease but was also widely used for cT3 recently. For patients with no SLN tumor cell involvement, ALND could be omitted (13). The incidence of lymphedema of the upper limb has declined dramatically and the patient QoL has improved. SLNB has also become a standard surgical procedure for clinically negative axillary lymph nodes breast cancer patients in our institute since early 2010s. Recent years, many scholars believed that ALND for patients with low burden positive SLNs might be omitted either, since non-sentinel lymph node (non-SLN) metastasis was very low for these patients (14, 15, 16, 17). However, the universal management of axillary lymph nodes in patients with positive SLNs was still unknown. Several clinical trials including American College of Surgeons Oncology Group (ACSOG) Z0011 and European Organisation for the Research and Treatment of Cancer (EORTC) 10,981–22023 AMAROS had demonstrated that axillary radiotherapy and ALND had comparable locoregional control and much lower morbidity among patients with limited SLN metastasis. However, most patients enrolled in these two trials had no additional positive axillary lymph nodes (non-SLNs) in the ALND group. Only 97 of 355 (27.3%) patients had additional non-SLNs metastasis, and 13.7% of patients undergoing ALND had 4 or more involved nodes in Z0011. 220 of 672 (33%) patients with additional positive non-SLNs nodes in AMAROS. Thus, more than two-thirds of the patients underwent ALND without a therapeutic benefit from this surgical procedure, and preserving fossa axillaris should be the optimal choice for these patients, which were adopted by many prestigious breast cancer center and guidelines. Nevertheless, the management and outcomes of these trials have also been being questioned by many medical colleagues, because the rate of non-SLN involvement among the patients with SLNs metastasis varied from 30% to 80% in different publications, which were much higher than Z0011 and AMAROS (18, 19, 20, 21). Therefore, it may be irrational to extend the conclusion of avoiding ALND based on Z0011 and AMAROS to different clinical scenarios without predicting the residual metastatic lymph nodes burdens. To more accurately make clinical decision, several studies have suggested using scoring systems or nomograms to predict the probability of non-SLN involvement in patients with at least one SLN metastasis (12, 22, 23). However, none of such findings has been adopted by international breast cancer guidelines. Consequently, for breast cancer patients with positive SLNs, ALND, to do or to omit remained a dilemma of choice (13). Here in this study, we reviewed female patients with operable cT1-3N0 invasive breast cancer who underwent ALND in our breast cancer center due to SLN metastasis and tried to evaluate the prevalence of non-SLNs metastasis among breast cancer patients with SLN metastasis in multiple clinical scenarios and its impact on clinical decision-making.

## Material and methods

We identified 892 female patients with operable cT1-3N0 invasive breast cancer who underwent ALND in our breast cancer center due to SLN metastasis from 2017 to 2023. Patients who received neoadjuvant chemotherapy were excluded. All patients underwent breast-conserving surgery (BCS) or modified radical mastectomy (MRM) in the light of the tumor characteristics, current breast cancer guidelines and the patient’s preferences. The SLNB was performed using the nano-carbon dye injection in the periareolar/intradermal location. About 10 min after the injection, all visible stained and nonstained lymph nodes were resected as SLNs by surgeons trained for SLN biopsy. All SLNs were assessed immediately via frozen section examination and subsequently paraffin-embedded for further pathological diagnosis. SLN metastases were defined as macro-metastasis (pN1, metastasis size >2 mm), micro-metastasis (pN1mi, metastasis size between >0.2 mm and ≤2 mm), or isolated tumor cells (ITCs) (pN0 [i+], metastasis size ≤0.2 mm) according to Eighth Edition of the American Joint Committee on Cancer (AJCC) Cancer Staging Manual (24). The patients with SLN metastasis including micrometastases but not ITCs (either frozen or paraffin-embedded sections identified) further underwent ALND. The number of metastatic and nonmetastatic lymph nodes in the SLNs and non-SLNs calculated separately. We extracted clinicopathological features of patients from the medical records. The tumor histopathologic type, tumor size, histological and nuclear grade, lymphovascular invasion (LVI), estrogen receptor (ER) status, progesterone receptor (PR) status, HER2 status and Ki67 index were recorded. Molecular subtype of breast cancer was defined based on the status of ER, PR, HER2 and Ki67 index. Tumors were classified hormone receptor (HR) positivity if either ER (+≥1%) or PR (+≥20%) was positive (25, 26). HER2 positivity was determined if immunohistochemistry (IHC) yielded 3+ or the *in situ* hybridization (FISH) amplification test was positive (27). TNM stage was categorized according to the AJCC Cancer Staging Manual, 8th Edition. The prevalence of non-SLN metastasis among different clinicopathological traits and its correlation with the number of positive SLNs were analyzed. The optimal clinical decision-making was generalized. This study was approved by the institutional review board ethics committee of our hospital.

## Statistical analysis

D’Agostino and Pearson normality test was applied to check the normal distribution when indicated. Unpaired t-Test was applied to evaluate the difference between the means of two groups. Variable distribution was evaluated using the Chisquare Test and followup Tukey’s Multiple Comparison Test among different groups and between every other group, respectively. Multivariate analysis was performed by multiple logistic regression. All statistical analyses were performed using Graphpad Prism version 9.0. The number of lymph nodes was presented in mean ± SEM (Standard Error of Mean). All tests were two sided, and *p* < 0.05 was considered statistically significant. In order to assess the predictive value of the multivariate logistic regression model, the ROC was plotted to calculate the AUC and evaluate the predictive power of the nomogram model. The area under the ROC curve (AUC) was used to measure model discrimination.

## Results

The patients’ median age was 50 years old, ranging from 25 to 80 years old. Most cases were invasive ductal carcinoma (IDC), accounting for 88.45%. Invasive lobular carcinoma (ILC) accounted for 4.49%, and other special infiltrative breast cancer accounted for 7.06%. The median tumor size was 2.6 cm, ranging from 0.5 to 6.8 cm. 211 (23.65%) of the patients had pT1 disease, 41 (4.60%) of the patients had pT3 disease, and the rest 640 (71.75%) had pT2 disease. 33 (3.7%) had a grade 1 tumor, 450 (50.45%) had a grade 2 tumor, 312 (34.98%) had a grade 3 tumor and the rest 97 (10.87%) had no tumor grade data. The prevalence of breast cancer molecular subtype was also analyzed. The proportions of luminal A (ER+/PR+/HER2-/Ki-67 low (≤14%)), luminal B1 (HR+/HER2−/Ki-67 high (>14%)), luminal B2 (HR+/HER2+), HER2 overexpression, triple negative breast cancer (TNBC) (HR-/HER2-) subtype were 22.09%, 38.57%, 12.11%, 14.35% and 12.89%, respectively. LVI was presented in 621 (69.62%) patients. The summary of patients’ clinicopathological characteristics was shown in Table 1.

**TABLE 1:** The summary of patients’ clinicopathological characteristics.

Characteristics	No. (proportion)
Age (y) median 50, range (25–80)	No. (proportion)
≤40	128 (14.35%)
41∼50	364 (40.81%)
50∼60	290 (32.51%)
>60	110 (12.33%)
Histopathologic type	No. (proportion)
IDC	789 (88.45%)
ILC	40 (4.48%)
Others	63 (7.06%)
pT stage	No. (proportion)
T1	211 (23.65%)
T2	640 (71.75%)
T3	41 (4.6%)
Tumor grade	No. (proportion)
1	33 (3.7%)
2	450 (50.45%)
3	312 (34.98%)
unknown	97 (10.87%)
LVI	No. (proportion)
absent	271 (30.38%)
1+	166 (18.61%)
2+	172 (19.28%)
3+	283 (31.73%)
Molecular subtype	No. (proportion)
Luminal A	197 (22.09%)
Luminal B1 (HER2−)	344 (38.57%)
Luminal B2 (HER2+)	108 (12.11%)
HER2 overexpression	128 (14.35%)
TNBC (HR-/HER2−)	115 (12.89%)

IDC: invasive ductal carcinoma; ILC: invasive lobular carcinoma; HR: hormone receptor; HER2: human epidermal growth factor receptor type 2; LVI: lymphovascular invasion; Luminal A: ER+/PR+/HER2−/Ki67 low (≤14%); Luminal B (HER2−): HR+/HER2−/Ki67 high (>14%); Luminal B (HER2+): HR+/HER2+; TNBC: triple negative breast cancer.

The median number of harvested SLN was 4, ranging from 1 to 12, and the median number of SLN+ was 2, ranging from 1 to 9. In general, over three-fourths of the patients had only one or two SLNs with cancer cell metastasis in all patients enrolled. The median number of additionally dissected lymph nodes (non-SLNs) during ALND was 14, ranging from 1 to 42, and the median number of non-SLN+ was only 1, ranging from 0 to 32. 388 (43.50%) patients had no extra metastatic non-SLNs. 306 (34.30%) had only 1 to 3 metastatic non-SLNs. 198 (22.20%) patients had over 3 metastatic non-SLNs. In summary, the median number of total LNs resected during SLNB and ALND was 18, ranging from 4 to 46, and the median number of LN+ was 3, ranging from 1 to 40. 548 (61.44%) patients were N1, 244 (27.35%) patients were N2, and 100 (11.21%) patients were N3, according to AJCC breast cancer staging manual, Eighth edition. The summary of the number of lymph nodes distribution during different surgical procedures was shown in Table 2.

**TABLE 2:** The number of lymph nodes distribution during different surgical procedures.

Characteristics	No. (proportion)
No. of SLN median 4, range (1–12)	No. (proportion)
1	30 (3.36%)
2∼5	654 (73.32%)
>5	208 (23.32%)
No. of SLN + median 2, range (1–9)	No. (proportion)
1	435 (48.77%)
2	242 (27.13%)
≥3	215 (24.10%)
No. of non-SLN median 14, range (1–42)	No. (proportion)
≤10	245 (27.47%)
10∼16	382 (42.83%)
>16	265 (29.71%)
No. of non-SLN + median 1, range (0–32)	No. (proportion)
0	388 (43.50%)
1∼3	306 (34.30%)
≥4	198 (22.20%)
No. of LN median 18, range (4–46)	No. (proportion)
<10	24 (2.69%)
10∼16	331 (37.11%)
>16	537 (60.20%)
No. of LN + median 3, range (1–40)	No. (proportion)
1∼3 (N1)	548 (61.44%)
4∼9 (N2)	244 (27.35%)
≥10 (N3)	100 (11.21%)

SLN: Sentinel lymph node resected during SLNB; non-SLN: none Sentinel lymph node resected during ALND; LN: total lymph nodes resected during SLNB, and ALND.

**FIGURE 1:**
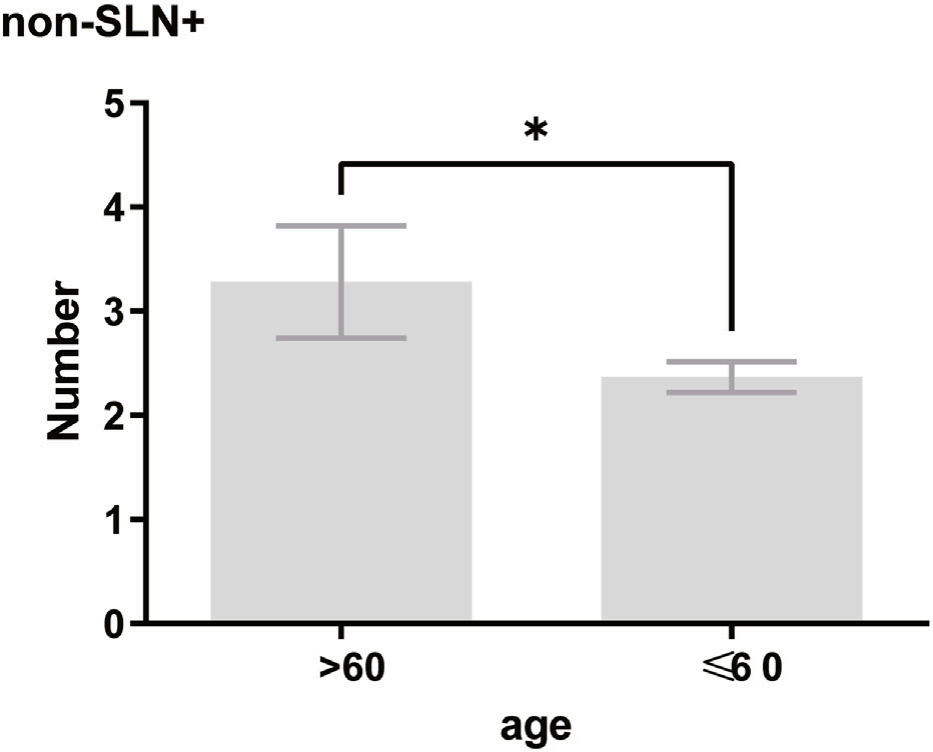
In univariate analysis, patients older than 60 years old showed a trend of relatively larger number (3.282 ± 0.541) of non-SLN + than that of younger than 60 years old (2.368 ± 0.144). Unpaired t-test, *p* = 0.0359.

**FIGURE 2:**
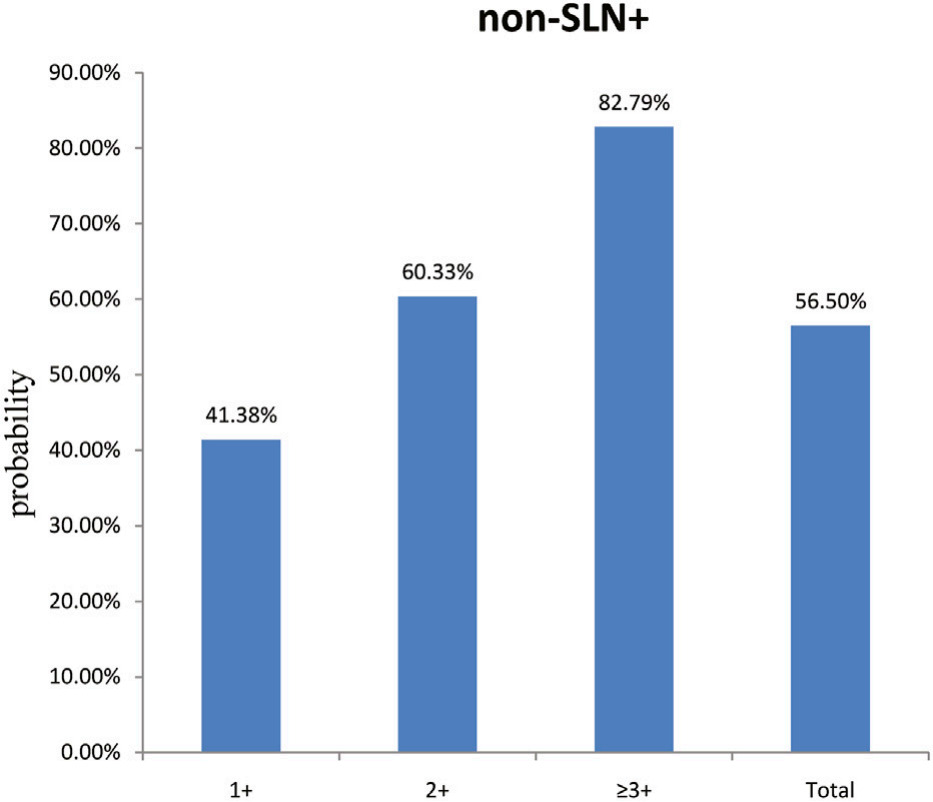
The probability of non-SLN + among patients with one, two and three or more SLNs+ was 41.38%, 60.33% and 82.79%, respectively. Overall, 56.50% of the patients had extra non-SLN metastasis.

**FIGURE 3:**
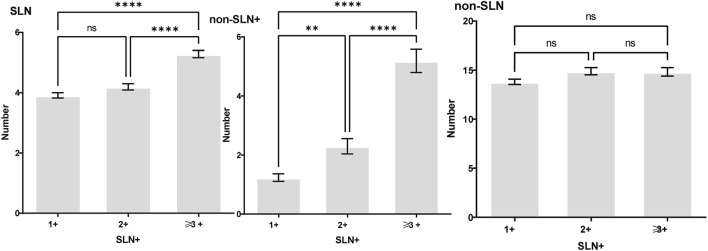
The number of SLNs+ was positively correlated with the number of SLN and non-SLN+, but not the number of non-SLN. The number of SLN and non-SLN + among patients with 3 or more SLNs+ was 5.284 ± 0.1197 and 5.191 ± 0.3924, more than that of patients with less than 3 SLNs+. Tukey’s multiple comparisons test, *p* < 0.05 was considered statistically significant.

**FIGURE 4:**
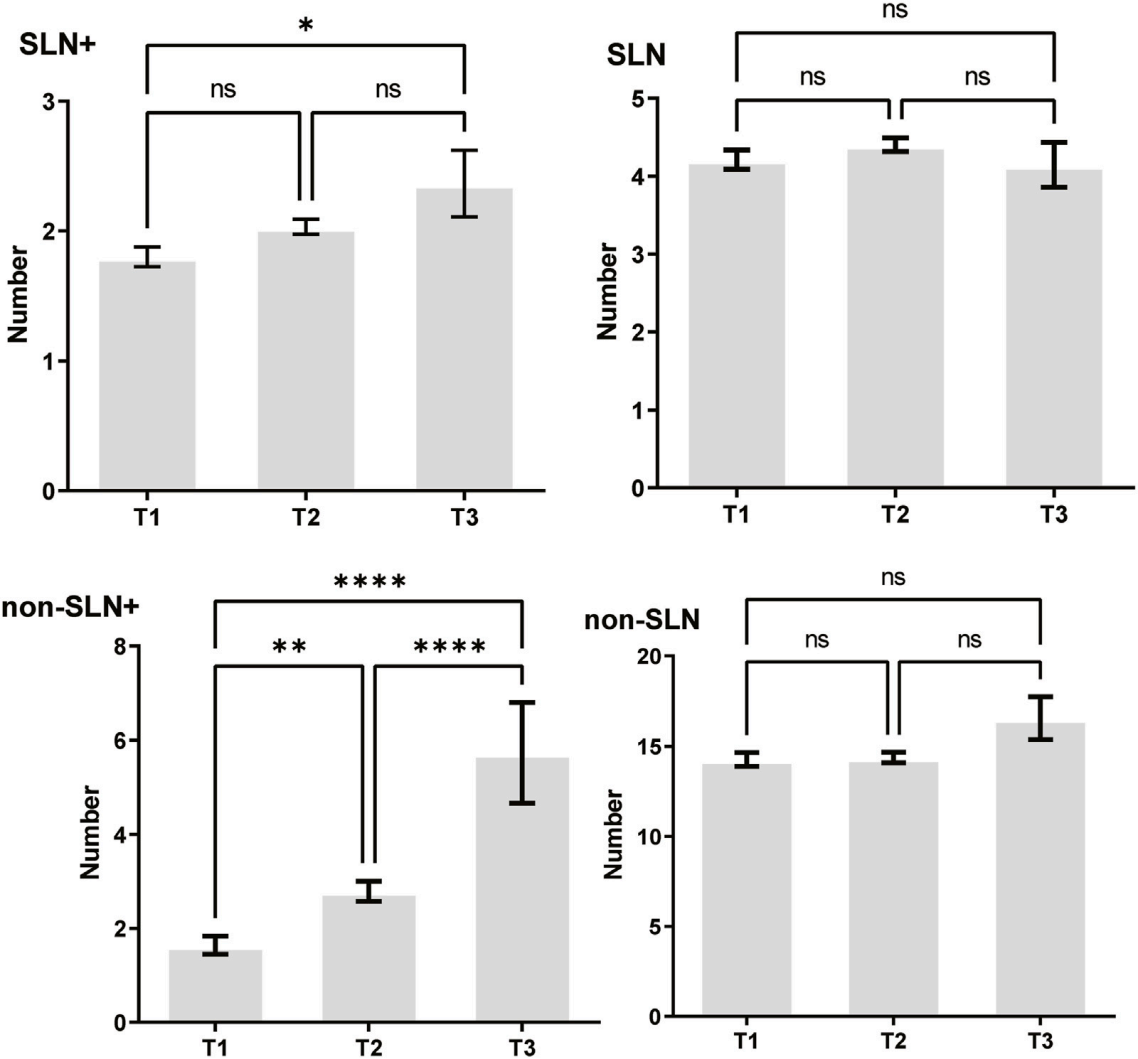
Tumor size also affected the number of SLNs+ and non-SLNs+, but not the number of SLNs and non-SLNs. The mean number of SLNs+ was 1.801 ± 0.076, 2.032 ± 0.058 and 2.366 ± 0.256 in T1, T2 and T3 patients respectively. The mean number of non-SLNs+ was 1.645 ± 0.192, 2.792 ± 0.213 and 5.732 ± 1.067 in T1, T2 and T3 patients respectively. Tukey’s Multiple Comparison between every other group.

**FIGURE 5:**
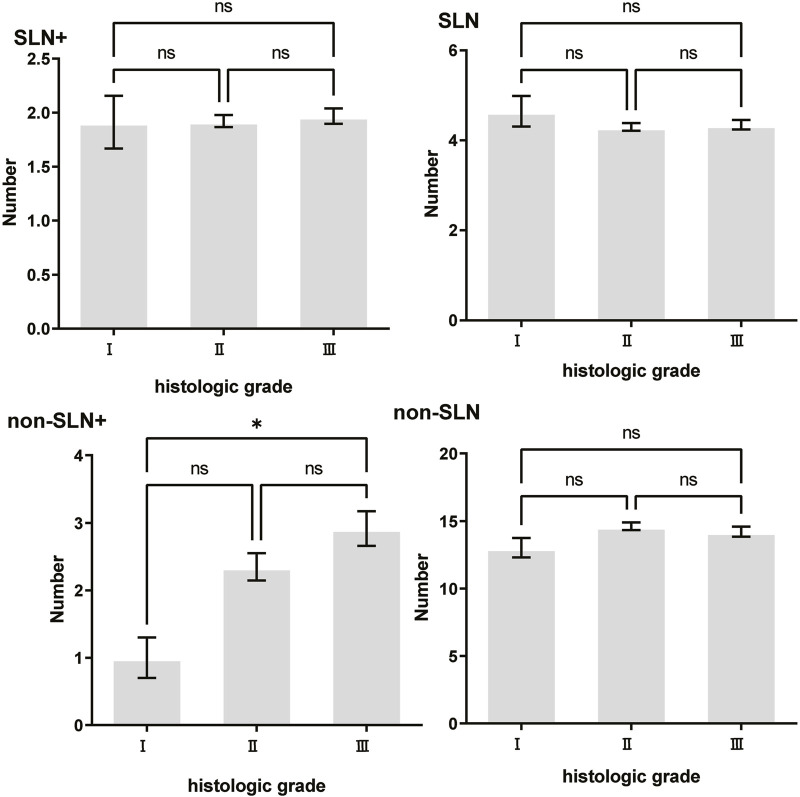
The impact of histologic grade on lymph node metastasis. Only the number of non-SLNs+ was largerin histologic grade III (2.927 ± 0.256) patients than histologic grade I (1.000 ± 0.302) patients, *p* = 0.038.

**FIGURE 6:**
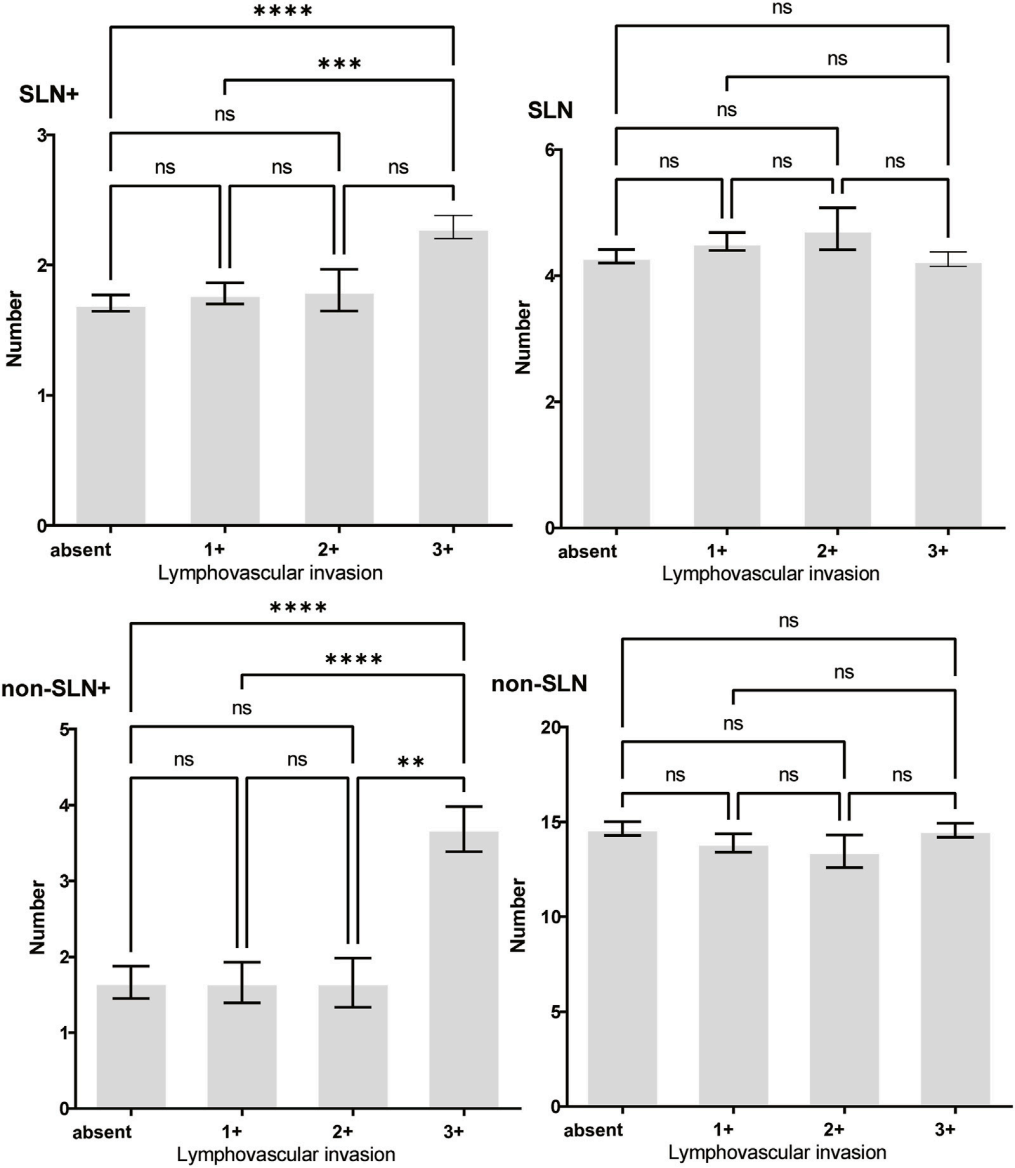
The impact of lymphovascular invasion (LVI) on lymph node metastasis. Both the number of SLNs+ and non-SLNs+ were larger in LVI 3+ patients than absent of LVI or 1+ patients.

**FIGURE 7:**
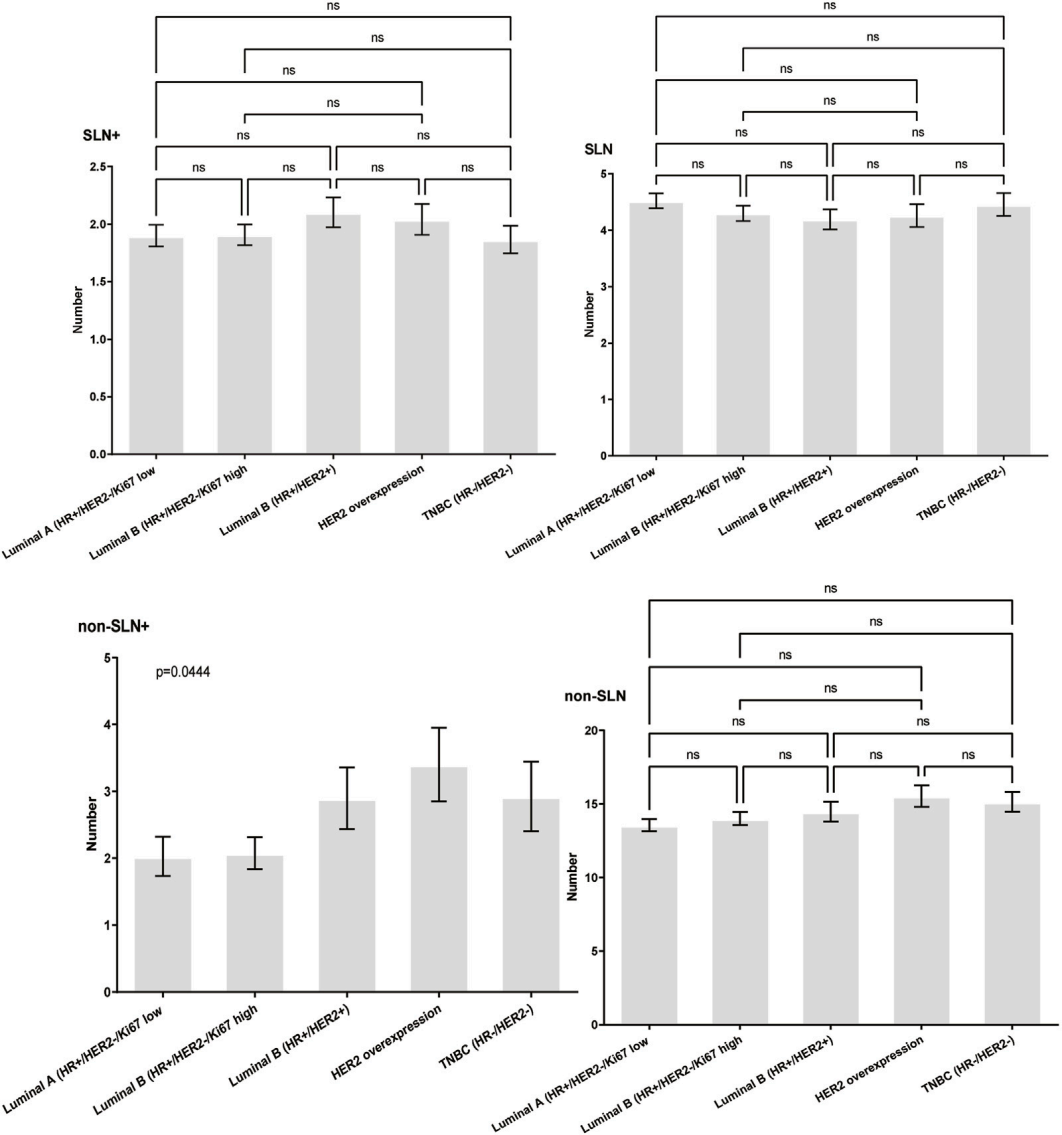
The impact of molecular subtypes on lymph node metastasis. The difference of non-SLNs + among different molecular subtypes showed statistical significance trend, using the Chisquare Test, *p* = 0.044.

**FIGURE 8:**
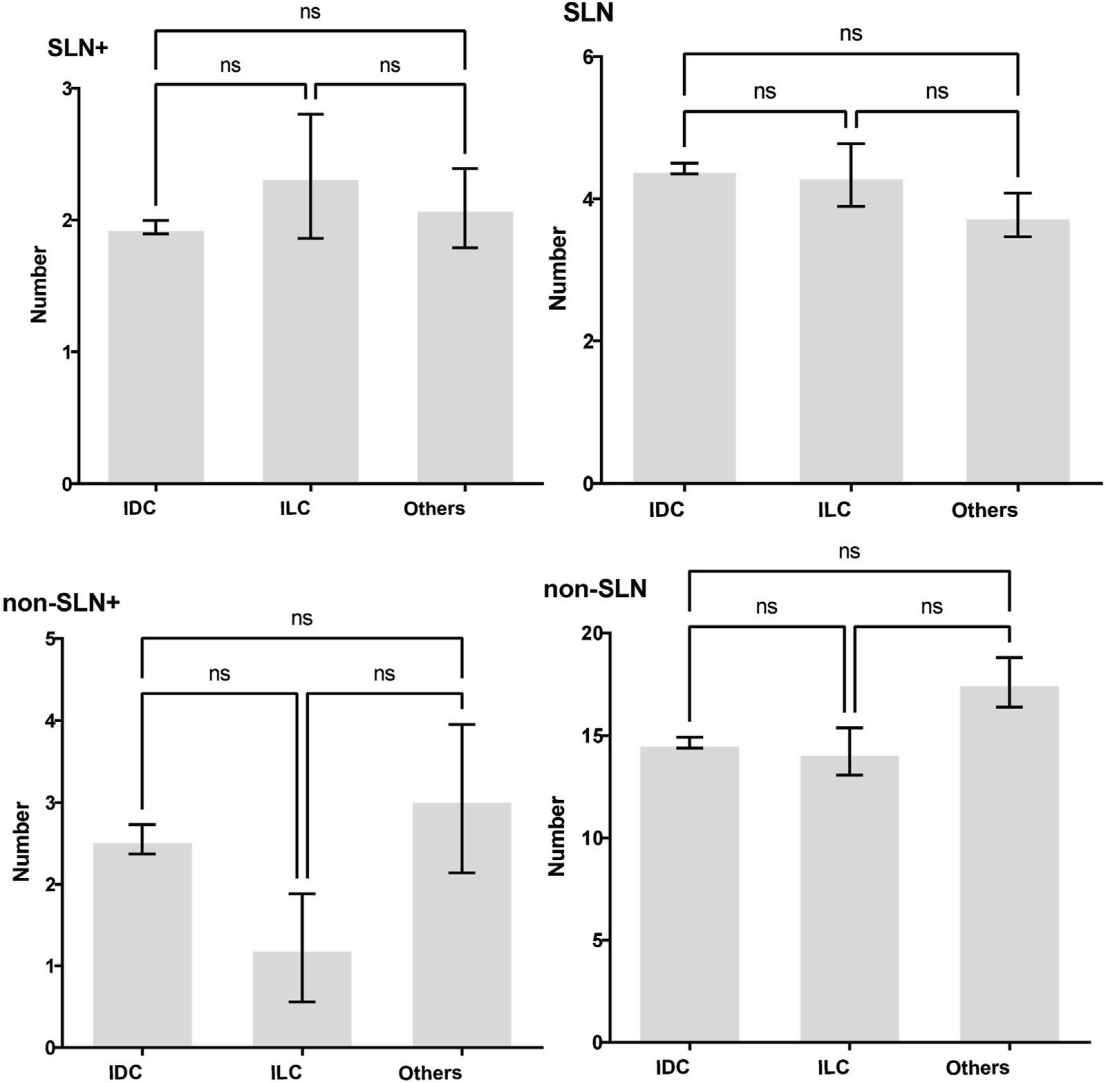
The impact of histopathologic type on lymph node metastasis. None of the number of SLN, SLN+, non-SLN and non-SLN+ was related with histopathologic type. Tukey’s Multiple Comparison between every other group, *p* > 0.05.

To clarify the main factors affecting non-SLNs metastasis, both univariate analysis and multivariate analysis were performed. We evaluated the number of non-SLN + among different clinicopathological traits, including patients’ age, the number of positive SLNs, tumor size, histologic grade, LVI and molecular subtypes. In univariate analysis, patients’ age may be a potential factor, patients older than 60 years old showed a trend of relatively larger number (3.282 ± 0.541) of non-SLN + than that of younger than 60 years old (2.368 ± 0.144), *p* = 0.0359, using unpaired t-test (Figure 1). The probability of non-SLN + among patients with one, two and three or more SLNs+ was 41.38%, 60.33% and 82.79%, respectively, 56.5% patients had positive non-SLN (Figure 2). The number of SLNs+ was positively correlated with the number of SLN and non-SLN+, but not the number of non-SLN. The number of SLN and non-SLN + among patients with 3 or more SLNs+ was 5.284 ± 0.1197 and 5.191 ± 0.3924, more than that of patients with less than 3 SLNs+ (Figure 3). Tumor size also affected the number of SLNs+ and non-SLNs+, but not the number of SLNs and non-SLNs. The mean number of SLNs+ was 1.801 ± 0.076, 2.032 ± 0.058 and 2.366 ± 0.256 in T1, T2 and T3 patients respectively. The mean number of non-SLNs+ was 1.645 ± 0.192, 2.792 ± 0.213 and 5.732 ± 1.067 in T1, T2 and T3 patients respectively, using Tukey’s Multiple Comparison between every other group (Figure 4). Only the number of non-SLNs+ was larger in histologic grade III patients than histologic grade I patients (Figure 5). Both the number of SLNs+ and non-SLNs+ were larger in LVI 3+ patients than absent of LVI or 1+ patients (Figure 6). The difference of non-SLNs + among different molecular subtypes showed statistical significance (Figure 7). None of the number of SLN, SLN+, non-SLN and non-SLN+ was related with histopathologic type (Figure 8).

To evaluate the risk factors influencing non-SLN spread among different clinicopathological traits by multivariate analysis, the patients’ age, tumor size, histologic grade, LVI, ER, PR, HER2 and Ki67 index, and the number of SLNs+, SLNs and non-SLNs were used. The number of SLNs+, SLNs and non-SLNs and LVI were identified as the independent factors for non-SLNs metastasis in the multivariate analysis (Table 3).

**TABLE 3:** Multivariate analysis for clinicopathological risk factors of non-sentinel lymph node metastasis.

Variable	OR	95% CI	*p*-value	Summary
Age (years)[≤60]	1.166	0.6748 to 2.036	0.5838	ns
pT staging [T2]	1.838	0.7062 to 5.472	0.2371	ns
pT staging [T1]	2.16	0.7980 to 6.610	0.1479	ns
ER [-]	0.8822	0.4908 to 1.586	0.6746	ns
PR [-/low]	1.239	0.7495 to 2.051	0.4031	ns
HER2 [-]	1.511	0.9453 to 2.432	0.0865	ns
Ki67 [low]	1.353	0.8237 to 2.225	0.2324	ns
No. of SLN+[2]	0.559	0.3672 to 0.8474	0.0064	**
No. of SLN+[≥3]	0.1522	0.0872 to 0.2577	<0.0001	****
No. of SLN [3∼5]	1.593	0.9429 to 2.720	0.0841	ns
No. of SLN [>5]	2.125	1.121 to 4.073	0.0217	*
No. of non-SLN [10∼16]	1.603	1.038 to 2.491	0.0342	*
No. of non-SLN [<10]	1.914	1.126 to 3.272	0.0169	*
Histologic grade [III]	0.5194	0.1735 to 1.462	0.2247	ns
Histologic grade [II]	0.6885	0.2428 to 1.823	0.4639	ns
LVI [absent]	2.612	1.682 to 4.083	<0.0001	****
LVI [1+]	2.149	1.300 to 3.566	0.0029	**
LVI [2+]	1.438	0.6584 to 3.101	0.3553	ns

OR: odds ratios; CI: confidence interval; ER: estrogen receptor, PR: progesterone receptor; HER2: human epidermal growth factor receptor type 2; SLN: sentinel lymph node; LVI: lymphovascular invasion.

**FIGURE 9:**
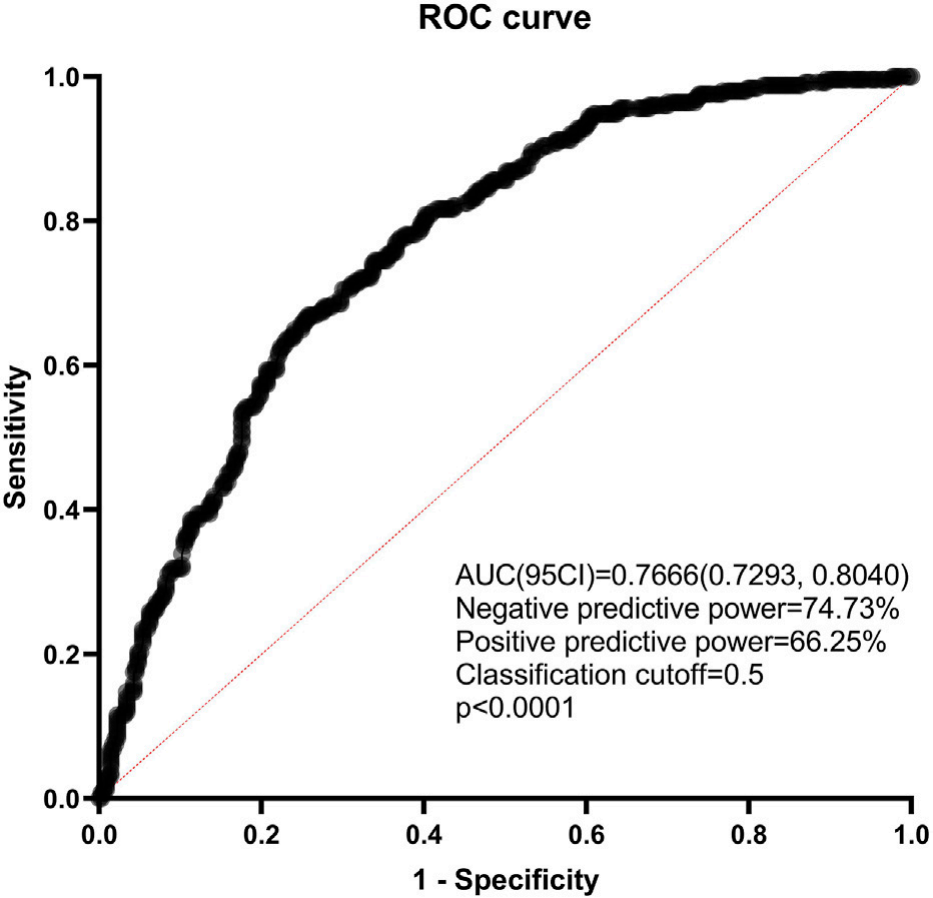
ROC curve of the combined with the estimated risk factors. ROC: receiver operating characteristics; AUC: areas under the ROC curve; CI: confidence interval.

The predictive nomogram found that the area under the ROC (receiver operating characteristic) curve was 0.7666 (95% confidence interval 0.7293–0.8040, *p* < 0.0001). Negative predictive power was 74.73%, while positive predictive power was only 66.25% (Figure 9). What’s more, the number of SLN metastases was the most significant predictive factor in both univariate and multivariate analysis.

## Discussion

Lymph node staging is essential for breast cancer treatment and prognosis. In recent two decades, this goal could be achieved through SLNB in clinically node negative breast cancer. The SLN has been considered as the first station of breast cancer metastasis, so SLNB may be a reliable procedure to evaluate lymph nodes spread, though there is a relatively high false negative rate even for the well-trained breast surgeons. For patients with no positive SLNs, ALND may be unnecessary. However, in the case of less than 3 positive SLNs, the properly clinical decision making is full of challenging (28, 29).

More and more publications in the last few years implied that ALND did not improve patients’ outcome, because the vast majority of the patients underwent ALND showed no additionally positive non-SLNs (30, 31, 32, 33, 34, 35). Consequently, how to precisely predict the probability of non-SLNs spread is essential. There are several different nomograms available to predict the risk of non-SLNs involvement in the presence of SLNs metastasis. Those tools commonly integrated patients’ clinicopathological traits including patient’s age, tumor size, histologic grade, LVI, ER, PR, HER2, Ki67 index and the number of SLNs + to evaluate the likelihood of non-SLNs metastasis, but they used a limited number of cases and lacked external validation (22, 36). The concept of total tumor load may accurately predict the status of non-SLNs and is another important tool for clinical decisions on early breast cancer patients. Nomogram to predict non-sentinel lymph node status using total tumor load determined by one-step nucleic acid amplification was first report from Thailand (37). Nevertheless, the optimal indication for ALND among the patients with a positive SLN still remains unclear.

In this study, 504 of 892 (56.50%) patients had extra metastasis in the non-SLN, the probability of non-SLN metastasis is roughly consistent with that in previous studies, but much higher than that of Z0011 and AMAROS trials. The number of SLNs+, SLNs and non-SLNs and LVI were predictive factors for non-SLN metastasis by multivariate analysis. Though the results of the Z0011 and AMAROS showed that the survival outcomes of SLNB plus radiotherapy were not inferior to those of ALND in breast cancer patients with limited SLNs metastasis and were cited by the American Society of Clinical Oncology Clinical Practice Guidelines, it is still too early to promote large-scale application among different breast cancer centers and ethnic groups since the non-SLN metastasis status varied significantly (from less than 30% to over 80%) and the regimen of radiotherapy including sites, dose and daily fraction was undefined. Whether axillary radiotherapy and ALND provides equivalent regional control in breast cancer patients with obvious residual metastatic lymph nodes in the axilla is still unknown.

In this context, there are massive studies conducted to predict non-SLN metastasis using scoring systems and nomograms. Because both preventive ALND and radiotherapy do not improve survival outcome but instead cause complications, accurately predicting the risk of non-SLN spread could be beneficial as it will help determine clinical decision making. Several nomograms integrating clinicopathologic factors such as tumor size, LVI, and positive and negative SLN metastases have been developed (38, 39). These nomograms found that the area under the ROC (receiver operating characteristic) curve was approximately 0.7 (40). What’s more, the number of SLN metastases was the most significant predictive factor in both univariate and multivariate analysis.

In the present study, we focused on the number of positive and total SLNs, the number of non-SLNs dissected and the LVI. Our study found that the negative predictive power of the nanogram was 74.73%, while positive predictive power was only 66.25%. Thus, the accurate prediction of non-SLN metastasis remains challenging to date. Our result suggests that clinicians should consider the risk of underestimating axillary lymph node metastases in patients who omitted ALND because even only 1 positive SLN did not ensure negative non-SLNs (41.38% probability with metastasis). Confirming negative non-SLNs in cases where the Z0011 criteria applied may help to avoid underestimating non-SLNs metastasis in certain clinical scenarios, but please do not assume that non-SLNs have no metastasis and omit ALND in patients with less than 3 positive SLNs. Whatever, ALND not only removed the potential metastatic lymph nodes but also provided decision-making basis for adjuvant CDK4/6 inhibitors treatment for luminal breast cancer. Since adjuvant CDK4/6 inhibitors (eg, abemaciclib) improve survival of luminal breast cancer at high risk, without ALND makes revealing four or more lymph nodes metastases impossible, which results in these patients not meeting the criteria of adjuvant CDK4/6 inhibitors therapy (41).

## Conclusion

To omit ALND in patients with higher tumor burden outside the Z0011 and AMAROS criteria should be considered with caution in clinical decision-making. ALND not only removed the potential metastatic lymph nodes but also provided more detailed lymph node staging for clinical adjuvant therapy, especially for CDK4/6 inhibitors usage in luminal breast cancer. To evaluate whether axillary radiotherapy and ALND provides equivalent regional control in breast cancer patients with obvious residual metastatic lymph nodes in the axilla, a well-matched prospective randomized controlled trial is an urgent need.

## Data Availability

The raw data supporting the conclusions of this article will be made available by the authors, without undue reservation.
